# Supplementary material for: Heritability of Gene Expression Measured from Peripheral Blood in Older Adults
Source: Genes (Basel). 2024 Apr 16;15(4):495. doi: 10.3390/genes15040495 (PMC11049887; doi:10.3390/genes15040495)
Supplement: Supplementary file 1 [file genes-15-00495-s001.zip › SI_1_Heritability_Blood Gene Expression_Older Adults_Revised_Version.pdf]

Supplementary Figures:

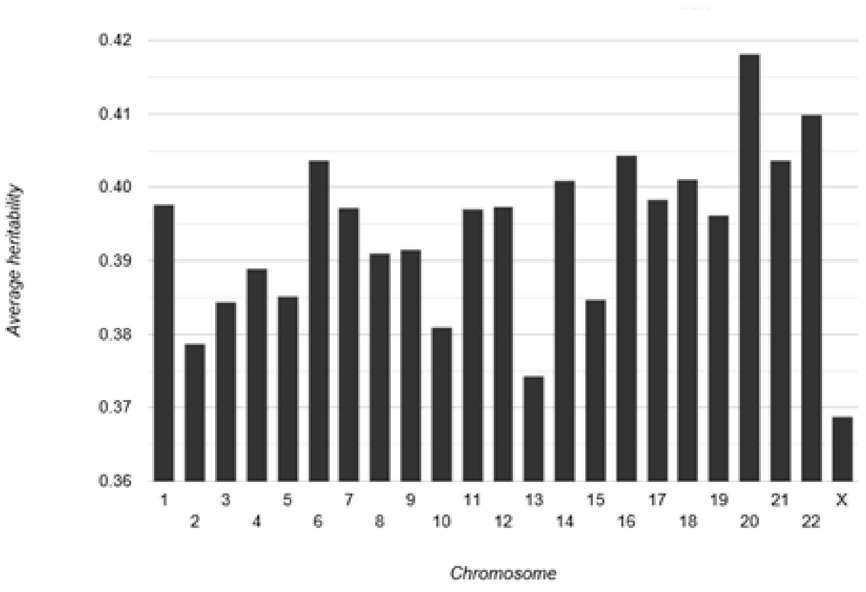

Figure S1: Average Heritability of Gene Expression across Chromosomes for the OATS cohort.

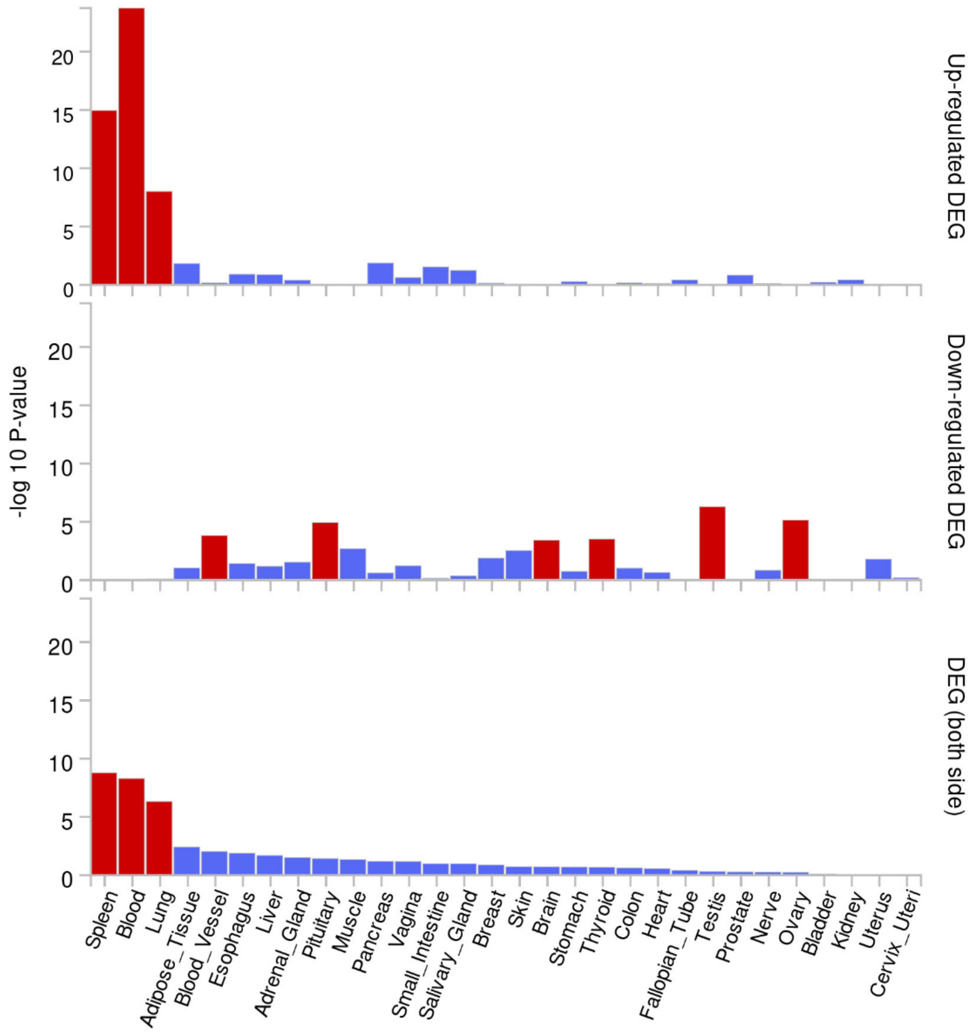

Figure S2: Tissue-specific gene expression levels of the OATS top 10% FDR significant genes across 30 different tissues using FUMA (based on GTEx). Red indicates FDR significant (FDR <0.05) gene expression; blue is not FDR significant.

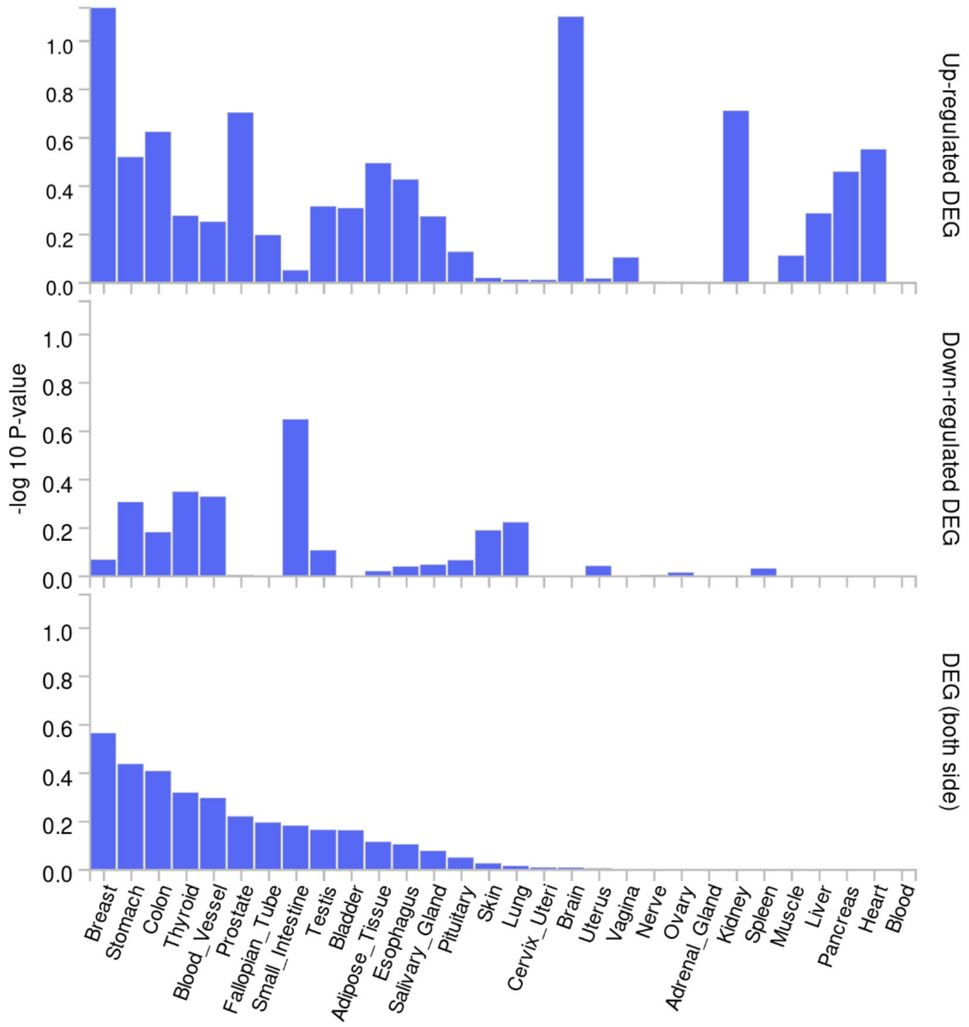

Figure S3: Upregulation and downregulation of the non-heritable gene set (n=460) for OATS across 30 different tissues using data from FUMA, showing that these genes are not significantly up or down regulated in any of the regions studied. Blue indicates the changes in gene expression are non-significant.

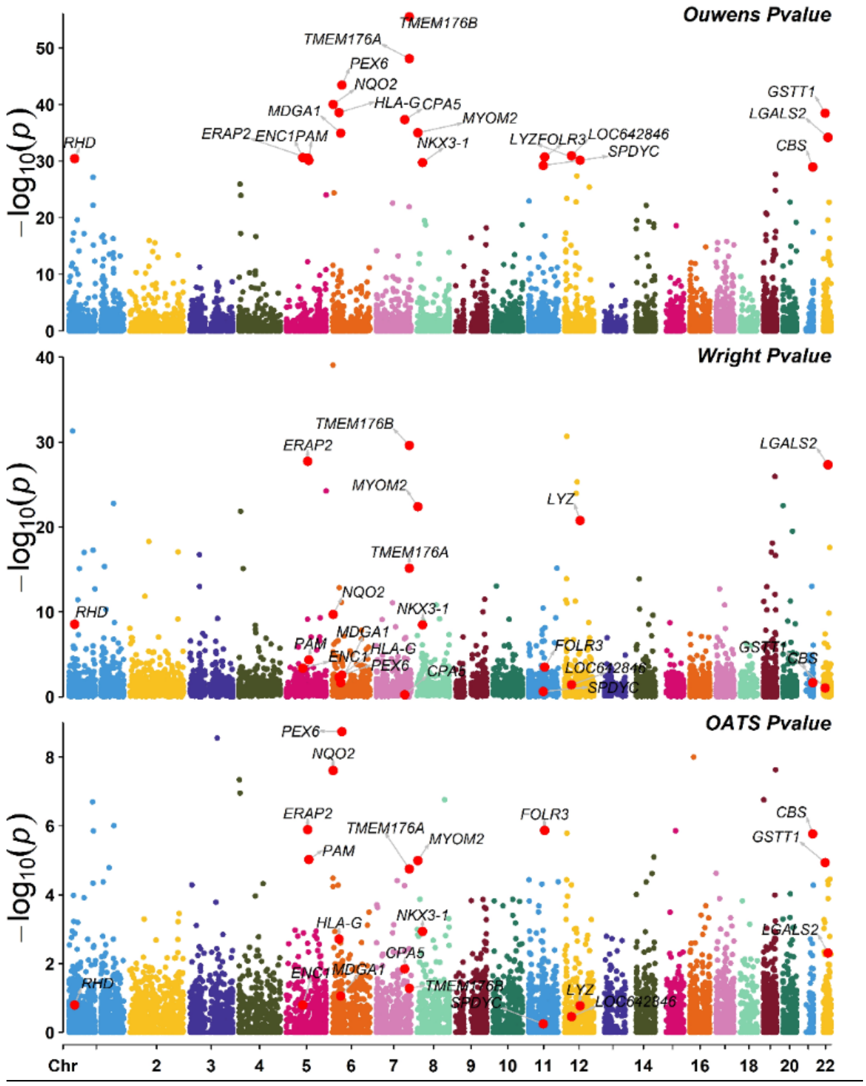

Figure S4: Manhattan plots for gene expression heritability for each of the three studies (OATS, Ouwens et al., 2020; Wright et al., 2014) using only the genes common to all cohorts (N= 9479). The y axis shows -log10(q-value). Gene names for Top 20 FDR significant genes displayed.

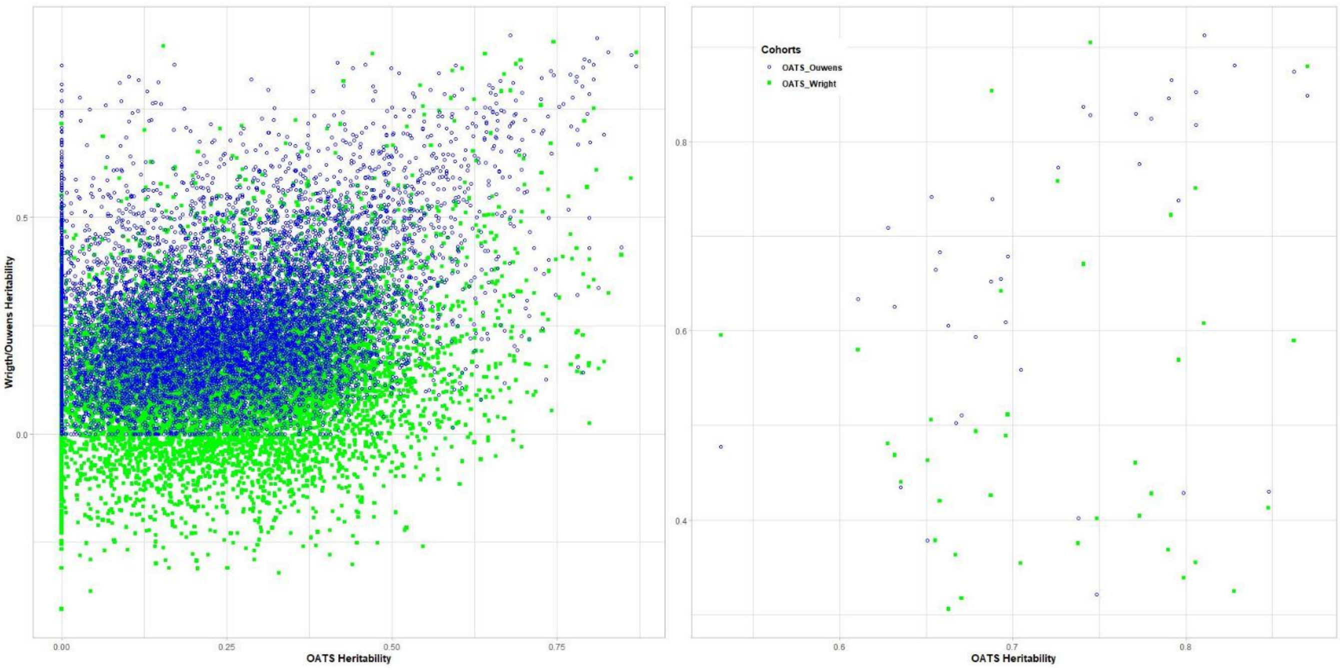

a b

Figure S5: Scatter plot comparing the gene expression heritability for the three studies (OATS, Ouwens et al., 2020; Wright et al., 2014) (a) using only the genes common to all cohorts (N= 9479); (b) using the genes overlapping between the three cohorts (n=38).

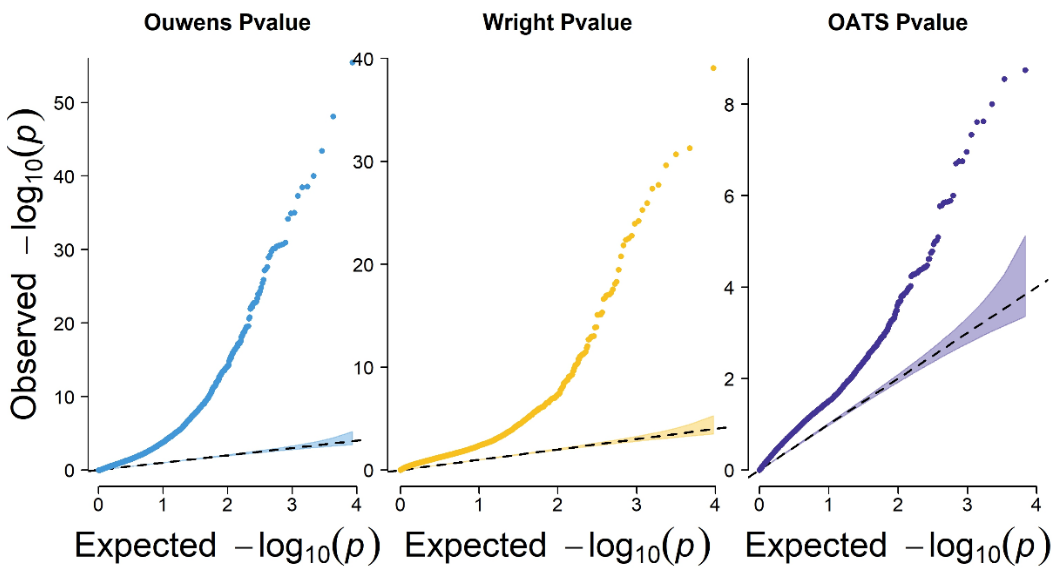

Figure S6: Scatter plot (QQ plot) for gene expression heritability for each of the three studies (OATS, Ouwens et al., 2020; Wright et al., 2014) using only the genes common to all cohorts (N= 9479). The y axis shows observed -log10 (p-value) and the x axis shows the expected -log10 (p-value).

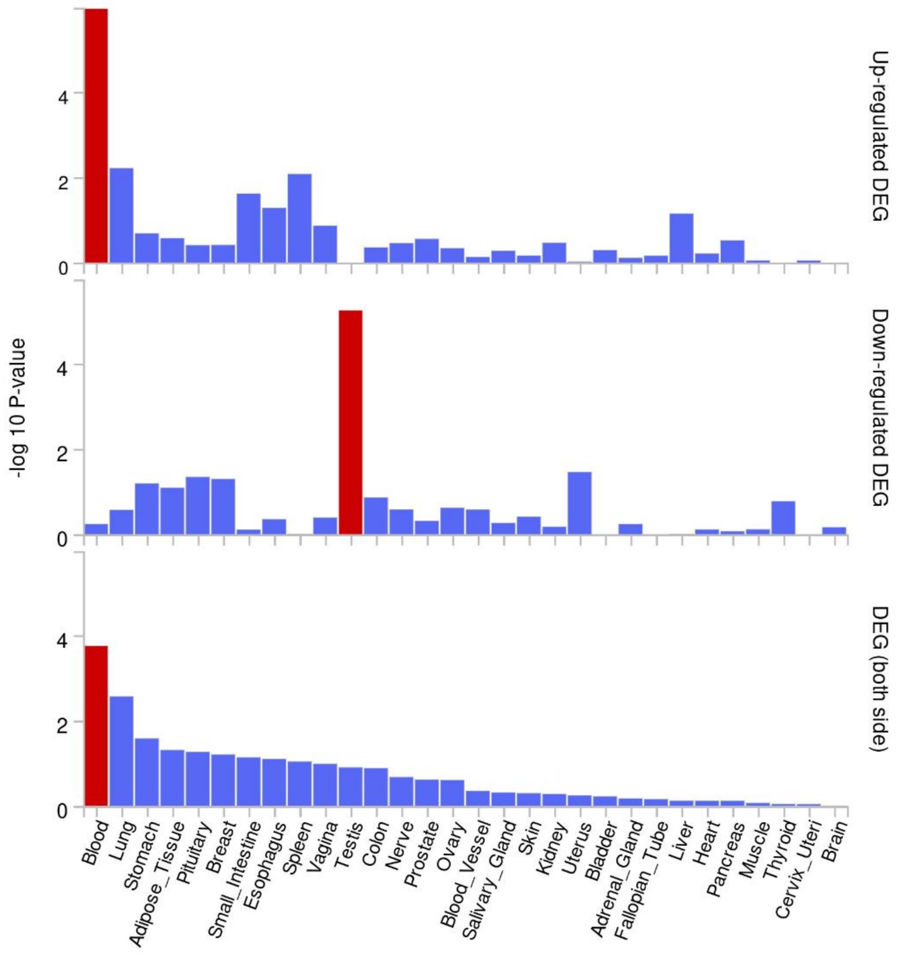

Figure S7: Tissue- specific gene expression levels of the FDR significant overlapping genes across the three studies (n=38) across 30 different tissues using FUMA (GTEx). Red indicates significant in gene expression, blue is not significant.
